# Integrative radiomics of intra- and peri-tumoral features for enhanced risk prediction in thymic tumors: a multimodal analysis of tumor microenvironment contributions

**DOI:** 10.1186/s12880-025-01790-2

**Published:** 2025-07-17

**Authors:** Liang zhu, Jiamin Li, Xuefeng Wang, Yan He, Siyuan Li, Shuyan He, Biao Deng

**Affiliations:** 1 Department of Cardiothoracic Surgery, Affiliated Hospital of Guangdong Medical University, Xiashan District, ZhanJiang, Guangdong Province China; 2 Guangzhou Medical University, Panyu District, Guangzhou, Guangdong Province China; 3 Guangdong Medical Universiy, Xiashan District, ZhanJiang, Guangdong Province China; 4 Department of Pharmacy of Shenzhen Kangning Hospital, Shenzhen Mental Health Center, Shenzhen Institute of Mental Health, Shenzhen, China; 5 Sun Yat-sen University, Yuexiu District, Guangzhou, Guangdong Province China; 6 Department of Radiology, Guangdong Women and Children Hospital, Guangzhou, China

**Keywords:** Thymic tumors, Intra- and peri-tumoral, Radiomics, Tumor microenvironment

## Abstract

**Objectives:**

This study aims to explore the role of intra- and peri-tumoral radiomics features in tumor risk prediction, with a particular focus on the impact of peri-tumoral characteristics on the tumor microenvironment.

**Methods:**

A total of 133 patients, including 128 with thymomas and 5 with thymic carcinomas, were ultimately enrolled in this study. Based on the high- and low-risk classification, the cohort was divided into a training set (*n* = 93) and a testing set (*n* = 40) for subsequent analysis.Based on imaging data from these 133 patients, multiple radiomics prediction models integrating intra-tumoral and peritumoral features were developed. The data were sourced from patients treated at the Affiliated Hospital of Guangdong Medical University between 2015 and 2023, with all imaging obtained through preoperative CT scans. Radiomics feature extraction involved three primary categories: first-order features, shape features, and high-order features. Initially, the tumor’s region of interest (ROI) was manually delineated using ITK-SNAP software. A custom Python algorithm was then used to automatically expand the peri-tumoral area, extracting features within 1 mm, 2 mm, and 3 mm zones surrounding the tumor. Additionally, considering the multimodal nature of the imaging data, image fusion techniques were incorporated to further enhance the model’s ability to capture the tumor microenvironment. To build the radiomics models, selected features were first standardized using z-scores. Initial feature selection was performed using a t-test (*p* < 0.05), followed by Spearman correlation analysis to remove redundancy by retaining only one feature from each pair with a correlation coefficient ≥ 0.90. Subsequently, hierarchical clustering and the LASSO algorithm were applied to identify the most predictive features. These selected features were then used to train machine learning models, which were optimized on the training dataset and assessed for predictive performance. To further evaluate the effectiveness of these models, various statistical methods were applied, including DeLong’s test, NRI, and IDI, to compare predictive differences among models. Decision curve analysis (DCA) was also conducted to assess the clinical applicability of the models.

**Results:**

The results indicate that the IntraPeri1mm model performed the best, achieving an AUC of 0.837, with sensitivity and specificity at 0.846 and 0.84, respectively, significantly outperforming other models. SHAP value analysis identified several key features, such as peri_log_sigma_2_0_mm 3D_firstorder RootMeanSquared and intra_wavelet_LLL_firstorder Skewness, which made substantial contributions to the model’s predictive accuracy. NRI and IDI analyses further confirmed the model’s superior clinical applicability, and the DCA curve demonstrated robust performance across different thresholds. DeLong’s test highlighted the statistical significance of the IntraPeri1mm model, underscoring its potential utility in radiomics research.

**Conclusions:**

Overall, this study provides a new perspective on tumor risk assessment, highlighting the importance of peri-tumoral features in the analysis of the tumor microenvironment. It aims to offer valuable insights for the development of personalized treatment plans.

**Clinical trial number:**

Not applicable.

**Supplementary Information:**

The online version contains supplementary material available at 10.1186/s12880-025-01790-2.

## Key points

• By using multimodal fusion and integrating both intra- and peri-tumoral features across different peri-tumoral ranges (1 mm, 2 mm, 3 mm), the study aims to improve the predictive accuracy of the model.

• SHAP value analysis was performed to assess the contribution of each feature to the model’s predictions, providing interpretability for individual features and enhancing the clinical applicability of the model.

**Supplementary Information:**

The online version contains supplementary material available at 10.1186/s12880-025-01790-2.

## Introduction

The tumor microenvironment plays a pivotal regulatory role in tumor initiation, progression, and metastasis [1, 2]. Radiomics, which extracts high-dimensional quantitative features from medical imaging, provides critical support for precise tumor diagnosis, treatment response evaluation, and prognosis prediction [3–5]. These features capture not only morphological information but also the dynamic interplay between tumors and surrounding tissues, offering profound biological insights for prognostic assessment and personalized therapeutic decision-making.

Thymoma [6], as a thymic epithelial tumor with poorly defined prognostic determinants, has historically depended on subjective imaging assessments that inadequately characterize its tumor microenvironment. This limitation becomes particularly significant given that while substantial advances have been made in thymoma risk stratification, existing research efforts have primarily concentrated on intratumoral characteristics, largely overlooking the systematic exploration of peri-tumoral heterogeneity and its underlying biological relevance. While peri-tumoral features have proven prognostic value in cancers like breast [7] and lung cancer [8] thymoma research still lacks quantitative radiomics analysis and dedicated model development for these regions. Although the study suggest that combined intra- and peri-tumoral features outperform intratumoral-only models, the independent contribution of peri-tumoral features—particularly their optimal boundary definitions—remains underexplored [9]. To bridge this gap, our study rigorously evaluates peri-tumoral features in thymoma risk stratification, offering novel insights into microenvironment dynamics and clinical prediction.

Building on this foundation, we construct multiple radiomics models to compare the predictive power of intra- versus peri-tumoral features. We specifically examine peri-tumoral expansion strategies (1 mm/2 mm/3 mm) to delineate their spatial influence on tumor behavior. To enhance clinical translatability, SHAP value analysis will elucidate model decision-making, ensuring both performance and interpretability [10].

## Materials and methods

### Study design

**Fig. 1:**
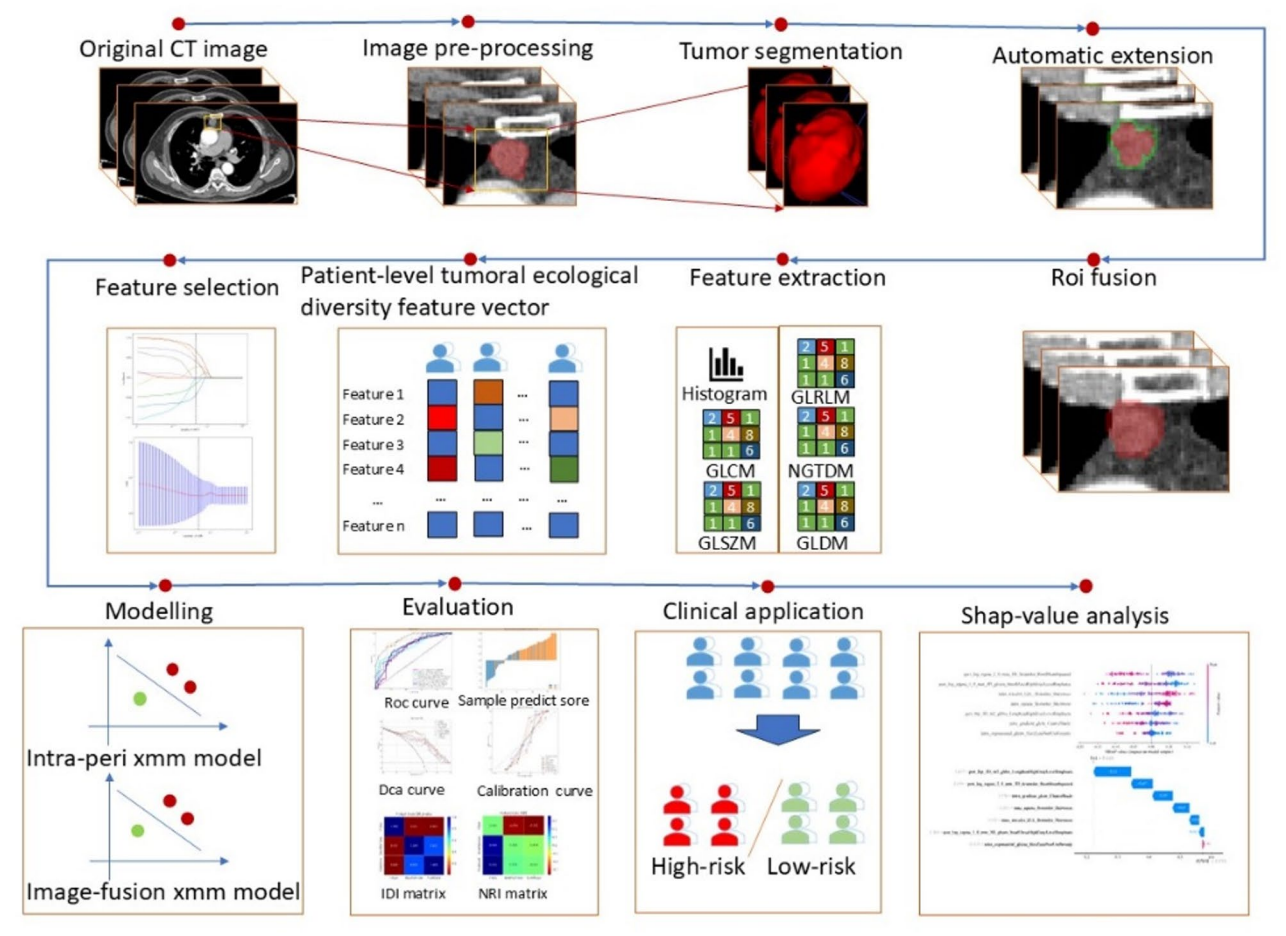
Research Flowchart

This retrospective study received approval from the Institutional Review Board (IRB) of the Affiliated Hospital of Guangdong Medical University. Given the retrospective study design, the IRB granted a waiver for informed consent. The study design and workflow are illustrated in Fig. 1.

**Fig. 2:**
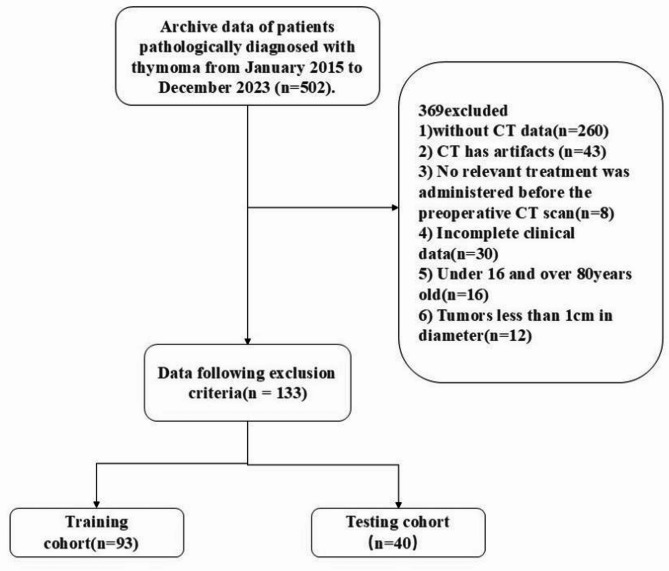
Patient Selection Flowchart

Patient data were retrieved from the hospital’s Picture Archiving and Communication System (PACS), comprising records for 128 patients diagnosed with thymoma and 5 patients with thymic carcinoma in Fig. 2. These records include cases treated at the hospital between 2015 and 2023, with a patient demographic of 74 males and 57 females, ages ranging from 16 to 80 years. The inclusion criteria were: (1) archived postoperative pathological diagnoses of thymoma dated between January 2015 and October 2023; (2) availability of complete CT images and clinical-pathological data. Exclusion criteria encompassed: (1) significant CT artifacts impacting image quality; (2) any prior treatment relevant to thymoma or thymic carcinoma before the preoperative CT scan; (3) incomplete clinical data.

All pathological diagnoses were independently verified by two experienced pathologists according to the World Health Organization (WHO) thymic tumor classification system. Based on established clinical criteria, tumors were stratified into: (1) low-risk groups (types A, AB, B1) and (2) high-risk groups (types B2, B3, and thymic carcinoma) [11, 12]. The final cohort comprised 133 patients (128 thymomas and 5 thymic carcinomas), who were randomly divided into training (*n* = 93) and testing (*n* = 40) sets at a 7:3 ratio. The baseline characteristics of each group are shown in Table 1.

This study divides imaging features into intra-tumoral and peri-tumoral characteristics, analyzing the performance of different thymoma and thymic carcinoma patients in intra-tumoral and peri-tumoral models across various pathological subtypes. The study design includes multiple fusion strategies, such as pre-fusion of intra- and peri-tumoral features (1 mm, 2 mm, 3 mm) and image fusion (1 mm, 2 mm, 3 mm), to evaluate the integration effects of intra- and peri-tumoral features.

**Table 1:** Baseline characteristics

feature_name	train-label = ALL	train-label = 0	train-label = 1	*p* value	test-label = ALL	test-label = 0	test-label = 1	*p* value
age	52.20 ± 14.21	55.23 ± 14.67	49.92 ± 13.55	0.075	55.90 ± 13.51	56.50 ± 14.84	54.79 ± 11.03	0.426
gender				0.01				0.457
0	45(48.39)	26(65.00)	19(35.85)		13(32.50)	10(38.46)	3(21.43)	
1	48(51.61)	14(35.00)	34(64.15)		27(67.50)	16(61.54)	11(78.57)	

### CT image acquisition

CT images of all patients were acquired using a multi-slice spiral CT scanner at the Affiliated Hospital of Guangdong Medical University, with the scanning range covering from the thoracic inlet to below the diaphragm, ensuring comprehensive coverage of the thymic tumors. The CT scan parameters were adjusted based on the patient’s body type and clinical requirements to ensure image quality. Specific parameters included: slice thickness and slice spacing set to 5 mm, with reconstruction adjusted to 1 mm; tube voltage set to 120 kVp, and tube current automatically adjusted according to the patient’s weight and the examination site, ranging from 150 to 250 mA; matrix size of 512 × 512; and soft tissue reconstruction algorithms to highlight the thymic tissue and tumor boundaries. All images were stored in the Picture Archiving and Communication System (PACS), and subsequent image analysis was performed based on the image data in PACS. To ensure the consistency and accuracy of radiomics feature extraction, all image data underwent standardization.

### ROI segmentation and automatic extension

In this study, a stringent dual-review protocol was implemented to ensure the accuracy of tumor segmentation. Manual delineation of the intra-tumoral region of interest (ROI) was performed by a radiologist with 5 years of clinical experience using ITK-SNAP software, under blinded conditions to minimize bias. Particular attention was given to precise boundary demarcation. All segmentations were subsequently reviewed by a senior radiologist with 20 years of expertise. In cases of discordance, the judgment of the more experienced radiologist was deemed conclusive.

After completing the intra-tumoral ROI segmentation, a custom Python algorithm was used to automatically expand the peri-tumoral region. Specifically, the peri-tumoral area was expanded outward from the intra-tumoral ROI boundary by 1 mm, 2 mm, and 3 mm, generating three peri-tumoral ROIs at different scales to capture imaging features of the tumor microenvironment. This expansion algorithm produced three independent peri-tumoral ROIs (i.e., intra- and peri-tumoral distinct ROIs) as well as three fused intra- and peri-tumoral ROIs, enabling stratified analysis of features at varying peri-tumoral distances.

This automatic expansion method significantly improved the efficiency and precision of peri-tumoral region extraction, standardizing and enhancing the reproducibility of the radiomics feature extraction process. By generating multiple peri-tumoral ROI layers at different distance levels, this approach established a rigorous data foundation for subsequent comparative analyses of intra- and peri-tumoral features. It also ensured the diversity of feature analysis and the reliability of research outcomes.

All CT images underwent standardized preprocessing to mitigate technical variability across scanners and acquisition years. Images were resampled to an isotropic 3 × 3 × 3 mm³ voxel grid using nearest-neighbor interpolation, with intensity normalization (scale factor: 1000) applied to minimize inter-scanner variations. Gray-level discretization used a fixed bin width of 5 HU with a + 1000 HU offset.

### Radiomics feature extraction and selection

**Fig. 3:**
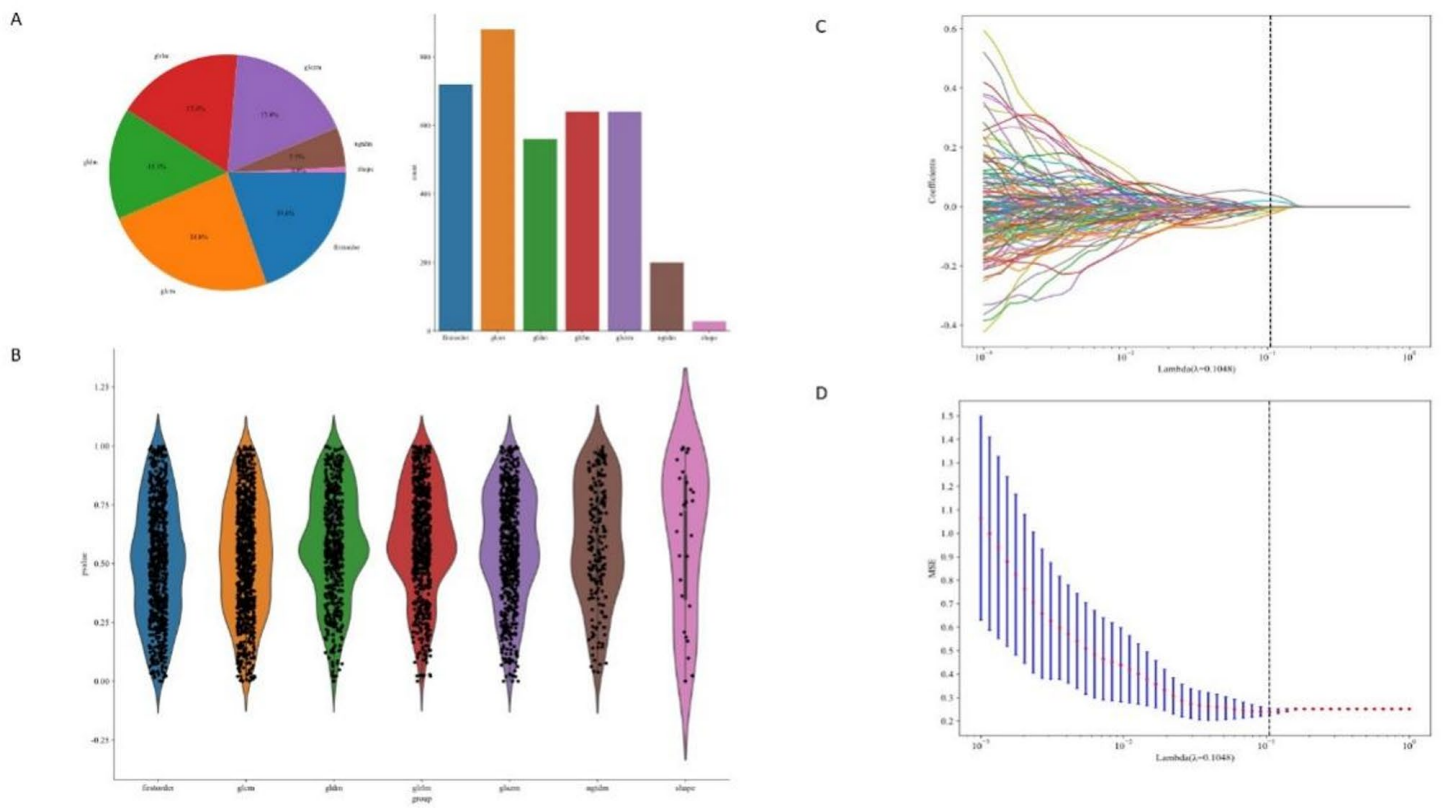
Radiomics Feature Type Distribution and feature selection. **A**. Proportion of Different Feature Types: This panel shows the proportion of various feature types among all features. **B**. Distribution of Different Features: This panel displays the distribution of the different features. **C**. LASSO Regression: Using 10-fold cross-validation, the optimal regularization weight λ is identified, minimizing the cross-validation error. **D**. Coefficient Path: This panel illustrates how the coefficients of different features shrink to zero as λ increases. The features corresponding to the optimal λ are retained for model construction

In this study, radiomics features were categorized into three main types: (I) first-order features, (II) shape features, and (III) higher-order features (Fig. 3A, B).

First-order statistical features reflect the symmetry, uniformity, and local intensity distribution variations of the measured voxels. These include metrics such as median, mean, and minimum values. Shape features quantitatively describe the geometric characteristics of the region of interest (ROI), such as tumor surface area, volume, and surface-to-volume ratio. These features help characterize the three-dimensional size and shape of the tumor. Higher-order features are derived from the original image or filtered images using different filtering methods and can reflect the spatial arrangement of voxel intensities within the image. They mainly include features extracted using methods such as Gray-Level Co-occurrence Matrix (GLCM), Gray-Level Run Length Matrix (GLRLM), Gray-Level Size Zone Matrix (GLSZM), and Neighboring Gray-Tone Difference Matrix (NGTDM) [13].

In this study, 1,835 hand-crafted features were extracted from each ROI. All features were extracted using a self-developed feature analysis program, based on the Pyradiomics library (http://pyradiomics.readthedocs.io).

To standardize the selected features, z-scores were applied. Feature selection was performed using the t-test with a p-value threshold of 0.05. To further reduce dimensionality and eliminate highly correlated features, the Spearman correlation coefficient was calculated for each pair of features. For feature pairs with a correlation coefficient greater than or equal to 0.90, only one feature was retained to reduce redundancy. In the feature analysis, hierarchical clustering [14] was performed on the Pearson correlation coefficient matrix of the preliminarily selected radiomic features using the `clustermap` function from the Seaborn library in Python. The correlation structure among features was visually presented through a heatmap and dendrogram, and highly correlated features were grouped into the same subclusters. The results are shown in Figure S1.We applied LASSO [15] regression for further feature selection to reduce redundancy and enhance model robustness. The selection process is illustrated in Fig. 3C and D, while the final selected features are presented in Figure S2.

After feature robustness and redundancy filtering, the remaining features demonstrated stability, predictive value, and non-redundancy.After feature robustness and redundancy filtering, the remaining features demonstrated stability, predictive value, and non-redundancy.

### Model construction and validation

#### Intra-periXmm model

In this study, we employed the LASSO algorithm for stringent feature selection (Fig. 3C, D), followed by the application of machine learning algorithms to construct radiomics risk models. The performance of each model was evaluated through comparative analysis, and multi-modal features were integrated on the basis of intra- and peri-tumoral feature fusion to explore the advantages of combining different imaging modalities in improving predictive accuracy. Here, “X” represents the distance of the peri-tumoral region. Three models were established: the intra-peri1mm, intra-peri2mm, and intra-peri3mm models.

#### ImagefusionXmm model

In this model, we applied the same rigorous feature selection process used for the intra-tumoral radiomics features and utilized the same machine learning algorithms to construct the final models, ensuring consistency and comparability between the intra-tumoral and peri-tumoral analyses. We established three models: imagefusion1mm, imagefusion2mm, and imagefusion3mm.

#### Model validation

In the model validation phase, we comprehensively assessed the predictive performance and discriminative ability of each model using multiple methods. First, receiver operating characteristic (ROC) curves were plotted, and the area under the curve (AUC) was calculated to quantify the classification ability of the models. Subsequently, the DeLong test was used to compare the AUC values of different models, evaluating their statistical differences in predictive accuracy. Additionally, Net Reclassification Improvement (NRI) [16] and Integrated Discrimination Improvement (IDI) [17] were employed to analyze the reclassification effectiveness and discriminatory power of the models, measuring the contribution of new features to model performance.

Furthermore, decision curve analysis (DCA) [15] was used to assess the clinical net benefit at different thresholds, validating the decision-making value of the models in real-world applications. Finally, calibration curves were plotted to evaluate the consistency between predicted probabilities and actual outcomes, testing the model’s calibration ability. The Hosmer-Lemeshow goodness-of-fit test was also performed. Detailed results can be found in Table 2.

**Table 2:** Performance of each model

Signature	Accuracy	AUC	95% CI	Sensitivity	Specificity	PPV	NPV	Cohort
INTRA	0.824	0.914	0.8572–0.9708	0.7	1	1	0.7	train
IntraPeri1mm	0.847	0.893	0.8257–0.9611	0.9	0.771	0.849	0.844	train
IntraPeri2mm	0.918	0.977	0.9475–1.0000	0.92	0.914	0.939	0.889	train
IntraPeri3mm	0.941	0.975	0.9434–1.0000	0.96	0.914	0.941	0.941	train
ImageFusion1mm	0.8	0.83	0.7415–0.9185	0.78	0.829	0.867	0.725	train
ImageFusion2mm	0.824	0.908	0.8470–0.9684	0.86	0.771	0.843	0.794	train
ImageFusion3mm	0.847	0.931	0.8803–0.9820	0.84	0.857	0.894	0.789	train
INTRA	0.684	0.702	0.5343–0.8688	0.692	0.68	0.529	0.81	test
IntraPeri1mm	0.842	0.837	0.6928–0.9811	0.846	0.84	0.733	0.913	test
IntraPeri2mm	0.605	0.662	0.4846–0.8385	0.769	0.52	0.455	0.812	test
IntraPeri3mm	0.711	0.715	0.5307–0.9000	0.692	0.72	0.562	0.818	test
ImageFusion1mm	0.658	0.685	0.5108–0.8584	0.692	0.64	0.5	0.8	test
ImageFusion2mm	0.658	0.789	0.6451–0.9333	0.923	0.52	0.5	0.929	test
ImageFusion3mm	0.632	0.658	0.4772–0.8398	0.615	0.64	0.471	0.762	test

### Statistical analysis

For continuous variables, either the t-test or Mann-Whitney U test was used for comparison based on their distribution characteristics. Categorical variables were analyzed using the chi-square test (χ² test).

All data analyses were performed using the Python 3.7.12 programming environment. Statistical tests were conducted using Statsmodels 0.13.2, radiomics feature extraction was carried out using PyRadiomics 3.0.1, and machine learning algorithms were implemented based on Scikit-learn 1.0.2.

## Results

### Clinical characteristics

The clinical characteristics of the patients are shown in Table 1. The table provides detailed information on the basic demographics of thymoma and thymic carcinoma patients, including variables such as gender, age, and others. Statistical analysis was performed to compare the distribution of characteristics between different patient groups, in order to evaluate the potential impact of these variables on tumor classification and prognostic prediction.

### Radiomics features analysis of intra-peri tumor

Using the Least Absolute Shrinkage and Selection Operator (LASSO) algorithm for radiomic feature selection, we ultimately identified seven key features from the intra-tumoral and peri-tumoral regions for model construction. These features, which pertain to tumor morphology, texture, and intensity distribution, effectively reflect tumor heterogeneity and its microenvironment characteristics. The LASSO algorithm reduces feature dimensionality through regularization, ensuring the robustness and efficiency of the selected features in the predictive model. These selected features exhibited high predictive power in subsequent model validation, providing strong support for risk prediction in thymoma and thymic carcinoma.

### Radiomics model comparison

**Fig. 4:**
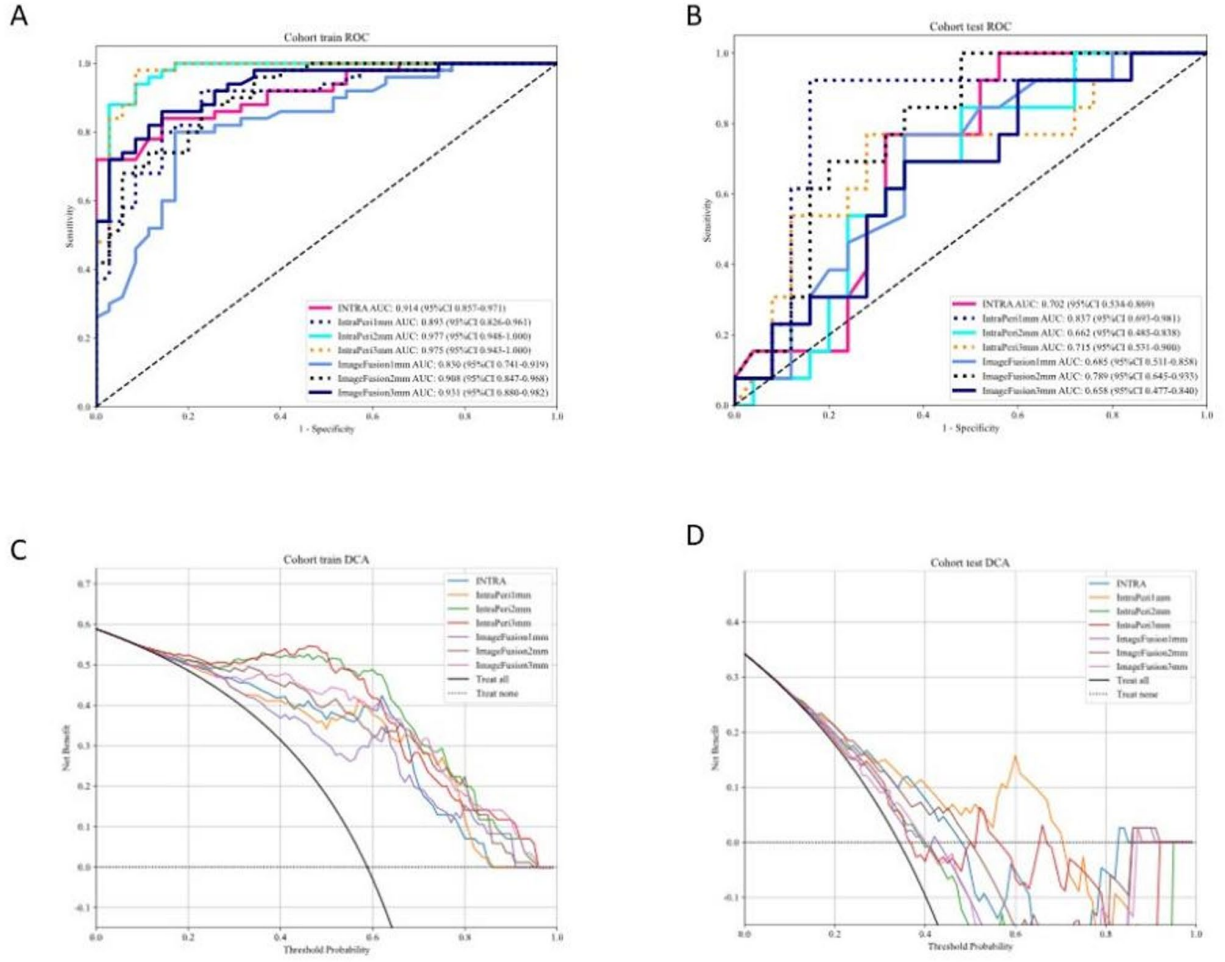
ROC Curves of All Models. **A**. Training Set ROC Curve: This panel presents the ROC curves for all models on the training set. **B**. Test Set ROC Curve: This panel presents the ROC curves for all models on the test set. **C**. DCA Curves for All Models: Decision Curve Analysis (DCA) for all models in the training set. **D**. DCA Curves for All Models: Decision Curve Analysis (DCA) for all models in the test set

As shown in Fig. 4A and B, ROC curves were plotted in the test set to demonstrate model performance. The IntraPeri1mm model exhibited the best performance with an AUC of 0.837, a sensitivity of 0.846, and a specificity of 0.84, indicating a significant enhancement in predictive power when incorporating features from the 1 mm peri-tumoral region. The INTRA model, with an AUC of 0.702, demonstrated relatively moderate performance, with sensitivity and specificity of 0.692 and 0.68, respectively, showing strong reliability in predicting positive cases. The IntraPeri2mm model had an AUC of 0.662, indicating overall poor performance. The IntraPeri3mm model, with an AUC of 0.715, sensitivity of 0.692, and specificity of 0.72, displayed a certain degree of balance but did not surpass the IntraPeri1mm model. Among the image fusion models, ImageFusion1mm and ImageFusion3mm had AUCs of 0.685 and 0.658, respectively, indicating limited predictive performance. The ImageFusion2mm model showed strong positive identification ability with an AUC of 0.789 and sensitivity of 0.923, but its specificity was only 0.52. Overall, the IntraPeri1mm model emerged as the best choice due to its high AUC and balanced sensitivity and specificity, while extensions to the peri-tumoral region beyond 2 mm and image fusion contributed limitedly to enhanced predictive power. As shown in Fig. 4C and D, Decision Curve Analysis (DCA) revealed that the IntraPeri1mm model provided the highest net benefit across clinical decision thresholds, supporting its practical applicability.

**Fig. 5:**
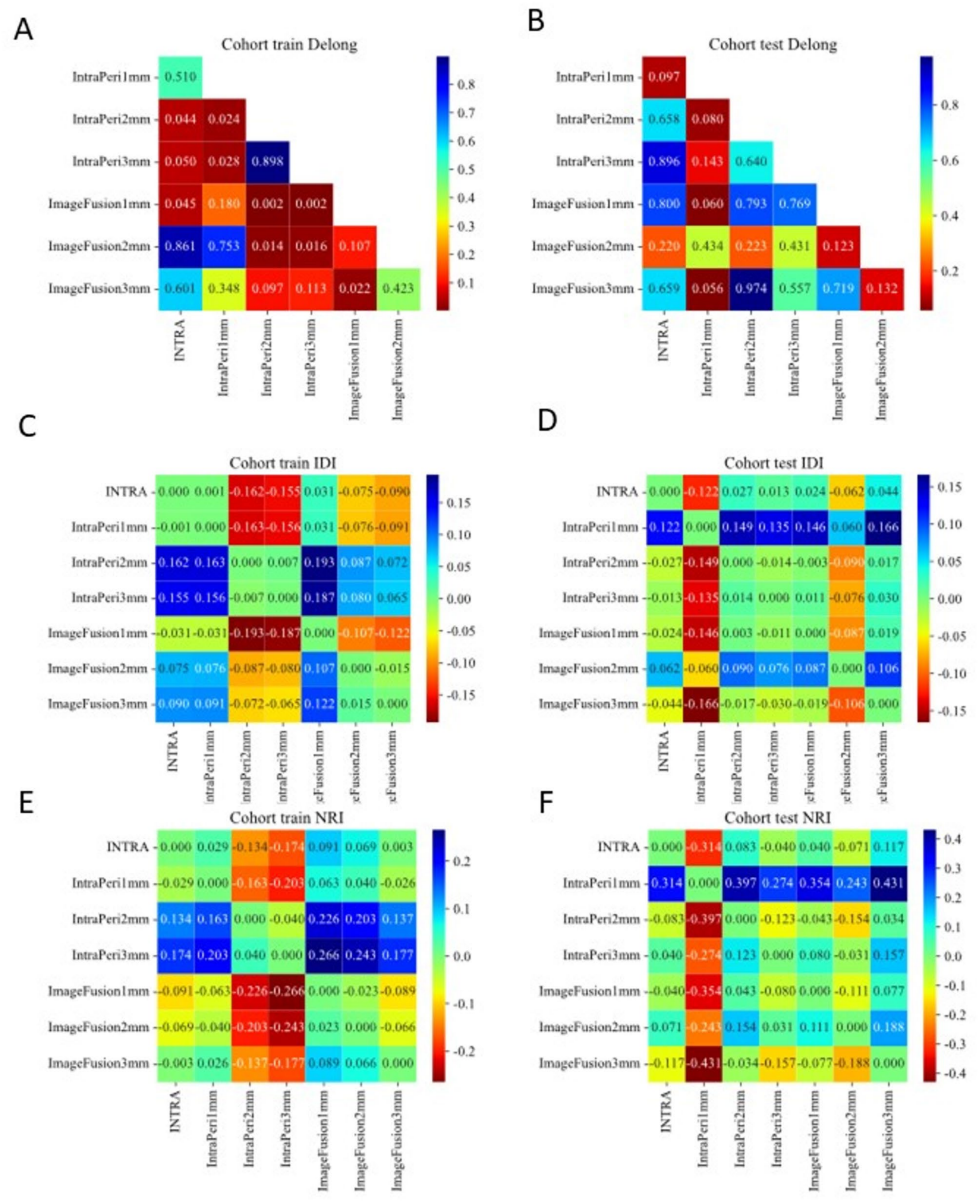
DeLong test, IDI test and NRI test comparing the performance of different models. **A**. DeLong Test for All Models: DeLong test results comparing the performance of different models on the training set. **B**. DeLong Test for All Models: DeLong test results comparing the performance of different models on the test set. **C**. IDI Test for All Models: IDI test results comparing the performance of different models on the training set. **D**. IDI Test for All Models: IDI test results comparing the performance of different models on the test set. **E**. NRI Test for All Models: NRI test results comparing the performance of different models on the training set. **F**. NRI Test for All Models: NRI test results comparing the performance of different models on the test set

**Fig. 6:**
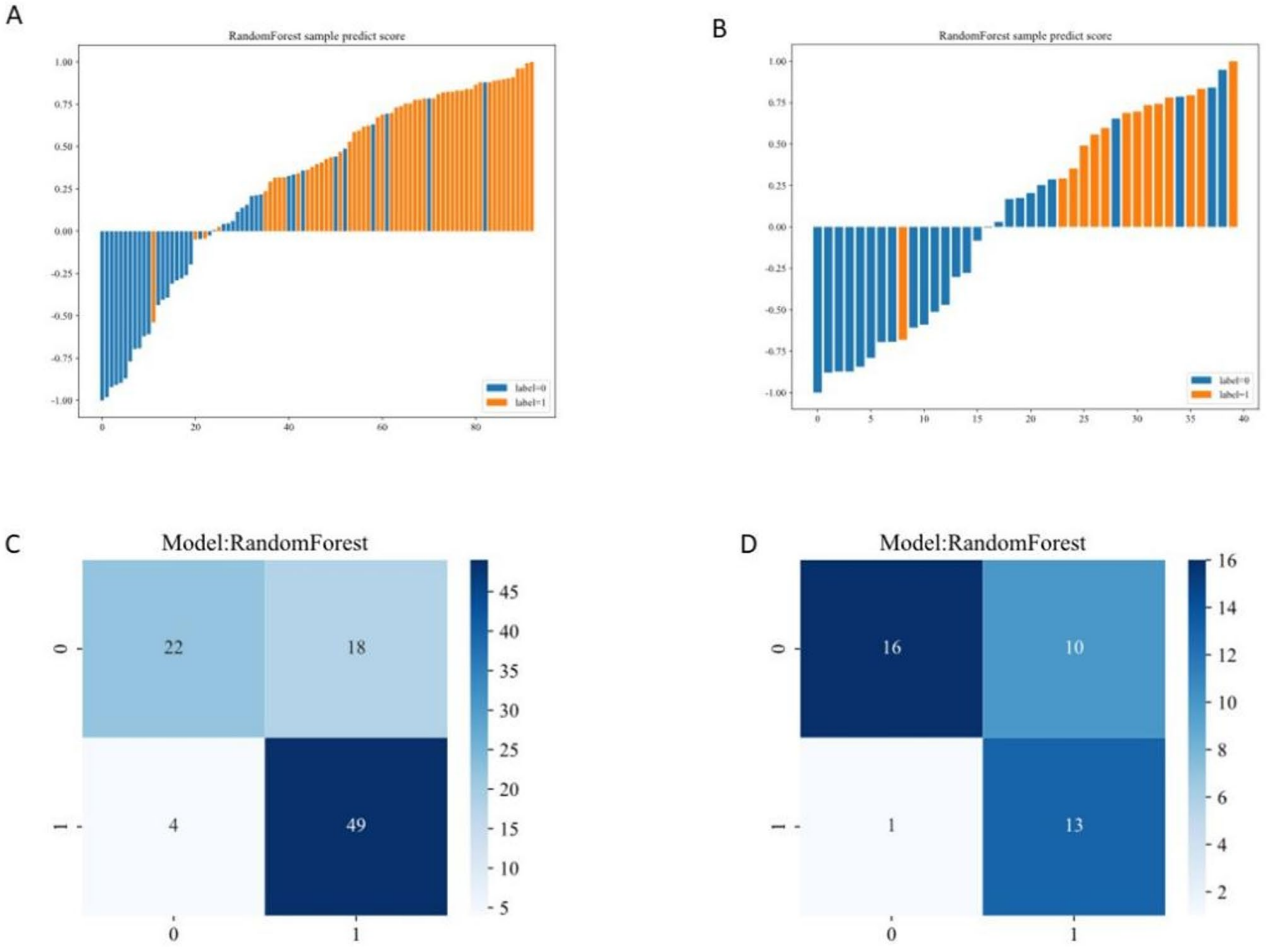
Waterfall Plot of intra-peri1mm Model and confusion matrix. **A**. Waterfall Plot of Habitat Model: Waterfall plot of the habitat model performance on the training set. **B**. Waterfall Plot of Habitat Model: Waterfall plot of the habitat model performance on the test set. **C**. Confusion matrix on the training set. **D**. Confusion matrix on the test set

The DeLong test confirmed significant differences between models (*p* < 0.05), with the IntraPeri1mm model consistently outperforming alternatives in Fig. 5. Further validation via Net Reclassification Improvement (NRI) and Integrated Discrimination Improvement (IDI) reinforced its superior predictive efficacy. A waterfall plot and confusion matrix were created to showcase the model’s effectiveness in Fig. 6. Calibration curves (Figure S3) were also provided.

### Shap-value analysis

**Fig. 7:**
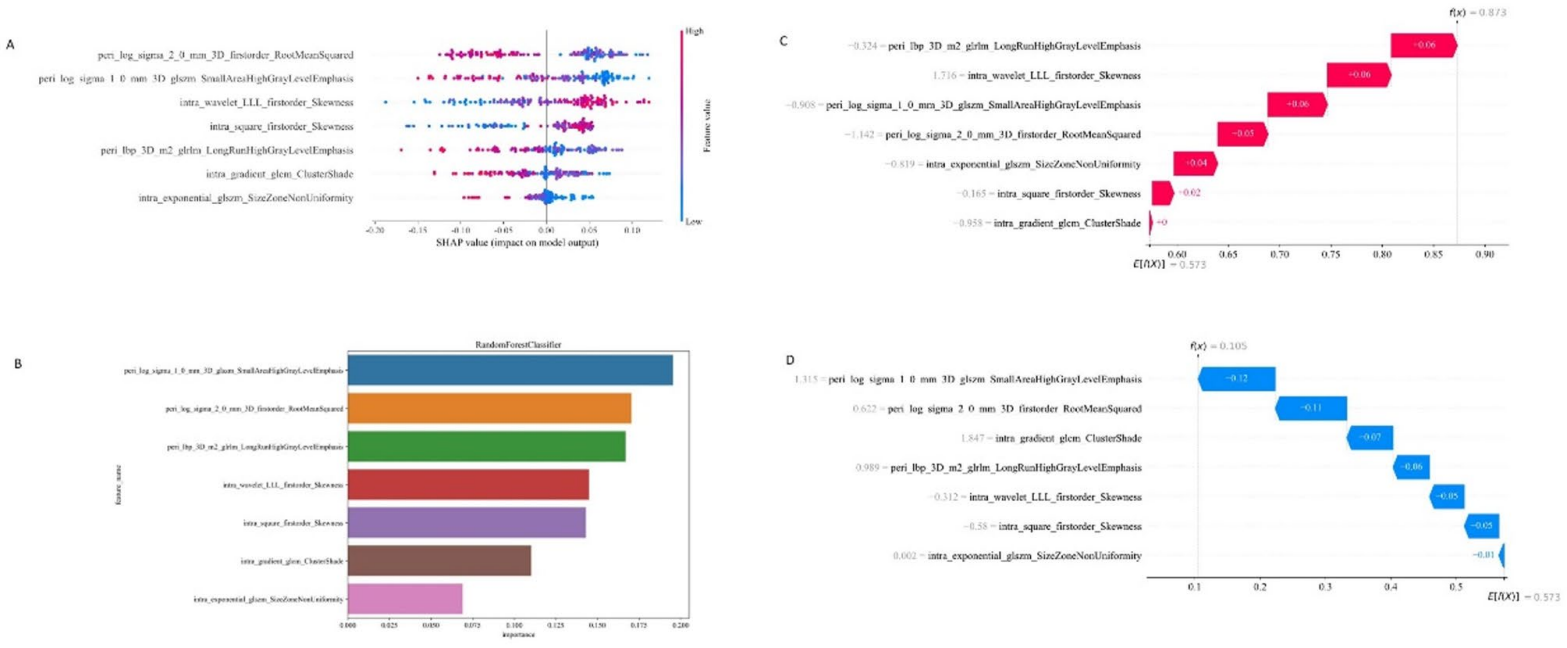
Model explanation. **A** SHAP value analysis. **B** Model important. **C** SHAP value analysis of sample 12. **D** SHAP value analysis of sample 7

SHAP (SHapley Additive exPlanations) value analysis is an effective method for model interpretability in Fig. 7. It quantifies the contribution of each feature to the model’s output, helping to understand the importance of each feature in the prediction process. In this study, we applied SHAP value analysis to the IntraPeri1mm model to reveal the driving factors behind its predictions.

By calculating the SHAP values for each feature, we were able to visually identify the features that have the greatest impact on the model’s predictions. These SHAP values provide insight into the positive or negative contribution of each feature to the final prediction, thus promoting transparency in the model’s decision-making process. Specifically, positive SHAP values indicate that an increase in the feature will raise the predicted risk, while negative values suggest that an increase in the feature will reduce the risk.

The analysis results indicate that certain geometric and texture features have a significant impact on prediction, as evidenced by their high SHAP values, which highlight their critical contributions to the model’s decision-making. Additionally, SHAP values helped identify lower-contribution features, supporting further feature selection and model optimization.

Several key radiomics features were identified as highly influential in tumor risk prediction. The peri_log_sigma_2_0_mm 3D_firstorder RootMeanSquared feature, which captures intensity distribution in the peri-tumoral area, showed a substantial contribution to risk prediction based on SHAP analysis. The peri_log_sigma_1_0_mm 3D_glszm SmallAreaHighGrayLevelEmphasis feature, reflecting the concentration of high gray levels in small areas, emerged as crucial in identifying high-risk tumor microenvironments.

Furthermore, intra_wavelet_LLL_firstorder Skewness provides a quantitative measure of intra-tumoral heterogeneity, with SHAP values indicating its significant contribution to the model’s predictions. The intra_square_firstorder Skewness feature, which quantifies the asymmetry in intra-tumoral intensity distribution, was also highlighted for its role in enhancing predictive capability.

In tumor edge identification, the peri_lbp_3D_m2 glrlm LongRunHighGrayLevelEmphasis feature was identified as influential, with SHAP analysis emphasizing its importance in this context. Similarly, intra_gradient_glcm ClusterShade, which captures cluster shadow effects in the intra-tumoral gray-level co-occurrence matrix, was shown to be vital for risk assessment. The intra_exponential_glszm SizeZoneNonUniformity feature, reflecting the non-uniformity of intra-tumoral region sizes, further underscored its value in the model.

We also illustrated the SHAP-based prediction process for two patients (Fig. 7C, D), providing an example of how SHAP values facilitate personalized interpretability, enhancing the transparency of individual predictions.

## Discussion

Recent studies have advanced thymoma risk stratification by leveraging radiomics and spatial heterogeneity analysis. For example, Liu et al. breast [18] developed a CT radiomics model to predict thymoma risk subgroups, while Liang et al. breast [19] used K-means clustering to segment tumor habitats, highlighting the role of spatial features. Yang et al. breast [20] further combined deep learning with intra-tumoral and habitat radiomics, demonstrating improved risk categorization. However, despite these advances, most thymoma studies prioritize intra-tumoral features, with limited systematic integration of peri-tumoral regions.

While preliminary investigations into tumor habitat and peritumoral imaging features have shown potential for thymoma risk assessment, a comprehensive approach integrating both intra- and peri-tumoral radiomics remains notably lacking breast [9, 21]. This gap persists despite evidence from other solid tumors demonstrating the enhanced predictive power of combined intra- and peri-tumoral feature analysis [22–24]. To address this limitation in thymoma research, our study specifically aims to develop an integrated radiomics model incorporating both intra- and peri-tumoral features for high- versus low-risk stratification. This approach seeks to establish a more robust framework for preoperative risk assessment, ultimately providing clinicians with more precise quantitative tools for therapeutic decision-making.

This study systematically evaluated the predictive value of intra- and peri-tumoral radiomic features for tumor risk assessment, with a particular focus on the biological and clinical implications of peri-tumoral microenvironment characteristics. Our results demonstrate that the IntraPeri1mm model, which incorporates peri-tumoral features within a 1 mm boundary, achieved superior predictive performance (AUC: 0.837) compared to models relying solely on intra-tumoral features. This finding not only reinforces the growing recognition of peri-tumoral regions as critical determinants of tumor behavior but also suggests that radiomic signatures at the tumor-stroma interface may capture early invasive or microenvironmental remodeling processes that are overlooked by conventional intra-tumoral analyses.

SHAP analysis revealed biologically interpretable high-impact features that substantiate the model’s predictive performance. The peri-tumoral heterogeneity feature (e.g., peri_log_sigma_2_0_mm_3D_firstorder_RootMeanSquared), reflecting coarse-scale intensity variations, likely corresponds to stromal desmoplasia or immune infiltration - pathological processes known to influence tumor aggressiveness and treatment resistance. Additionally, focal high-gray-level zones in peri-tumoral regions (e.g., peri_log_sigma_1_0_mm_3D_glszm_SmallAreaHighGrayLevelEmphasis) may represent microcalcifications, fibrotic changes or angiogenic hotspots, which have been established as imaging biomarkers of metastatic potential across various malignancies. Importantly, intra-tumoral skewness (e.g., intra_wavelet_LLL_firstorder_Skewness) demonstrated significant predictive value, where asymmetry in tumor intensity distribution may reflect necrotic cores, hemorrhage, or other structural disorganization characteristic of high-grade malignancies. Together, these radiomic signatures spanning both intra- and peri-tumoral domains provide compelling biological plausibility for the model’s discriminative capacity in thymoma risk stratification.

The IntraPeri1mm model’s robustness was further supported by NRI/IDI analyses and decision curve analysis (DCA), which confirmed its net benefit across clinical thresholds. The DeLong test validated its statistical superiority over comparator models. These results align with growing evidence that peri-tumoral regions harbor biologically meaningful information, potentially improving risk stratification beyond conventional intra-tumoral assessments.

However, this study also has certain limitations. First, the relatively small sample size may affect the generalizability of the model. Second, the retrospective design could introduce bias, potentially impacting the reliability of the results. Future research should consider prospective validation with a larger sample size to improve the external validity of the findings. Additionally, the variability in imaging data and differences in acquisition methods may affect model performance, which should be addressed in subsequent studies.

In summary, this study provides new evidence for risk prediction in thymoma and thymic carcinoma, laying a foundation for the exploration of personalized treatment strategies. We hope our research will stimulate further investigation and progress in oncology.Overall, this study offers a fresh perspective on tumor risk assessment, emphasizing the importance of radiomics features, particularly peri-tumoral features, in analyzing the tumor microenvironment. These findings are expected to provide valuable support for clinicians in developing personalized treatment plans.

## Electronic supplementary material

Below is the link to the electronic supplementary material.

Supplementary Material 1

Supplementary Material 2

Supplementary Material 3

Supplementary Material 4

## Data Availability

The datasets analyzed in this study are not publicly available due to patient privacy restrictions. De-identified data may be shared upon reasonable request to the corresponding author, subject to approval of a formal data access agreement ensuring ethical use and confidentiality.

## Abbreviations

AUC: Area Under the Curve
DCA: Decision Curve Analysis
IDI: Integrated Discrimination Improvement
LASSO: Least Absolute Shrinkage and Selection Operator
NRI: Net Reclassification Improvement
ROC: Receiver Operating Characteristic
ROI: Region of Interest
SHAP: SHapley Additive exPlanations
