# Supplementary figures and images for: Integrative radiomics of intra- and peri-tumoral features for enhanced risk prediction in thymic tumors: a multimodal analysis of tumor microenvironment contributions
Source: BMC Med Imaging. 2025 Jul 17;25:286. doi: 10.1186/s12880-025-01790-2 (PMC12272994; doi:10.1186/s12880-025-01790-2)

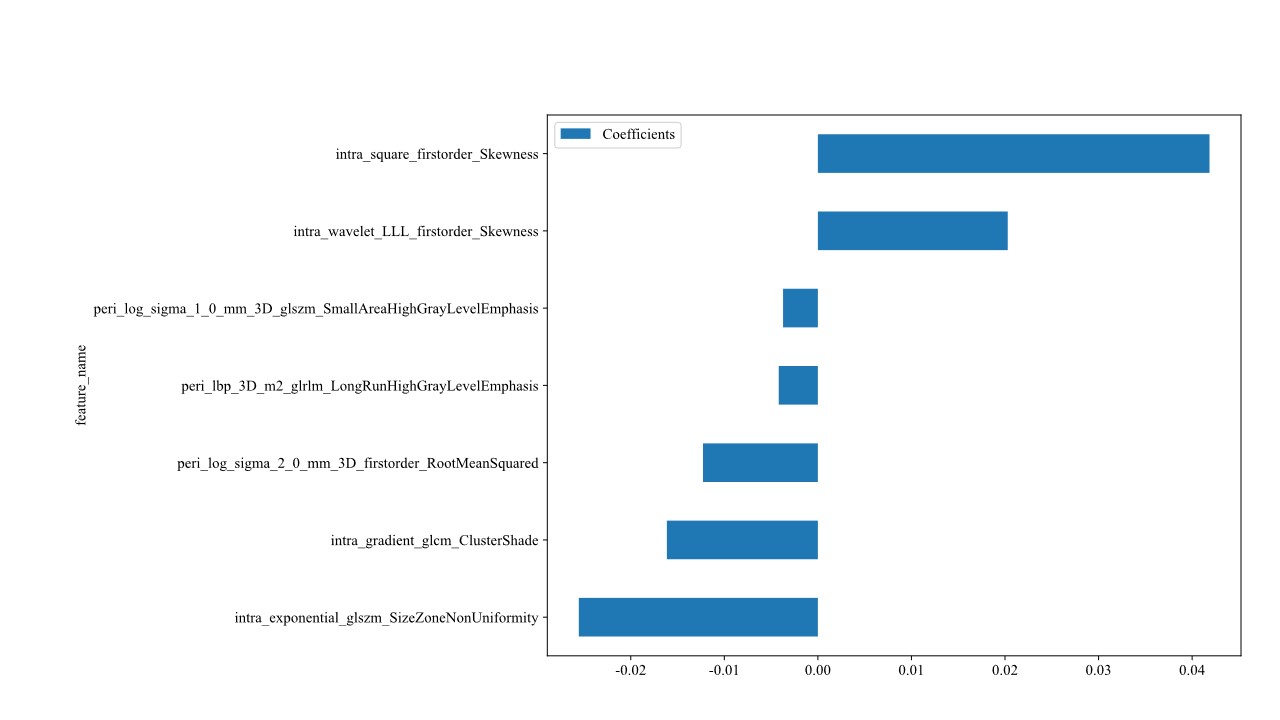

Supplement: Supplementary file 2 — Supplementary Material 2 [file 12880_2025_1790_MOESM2_ESM.jpg]

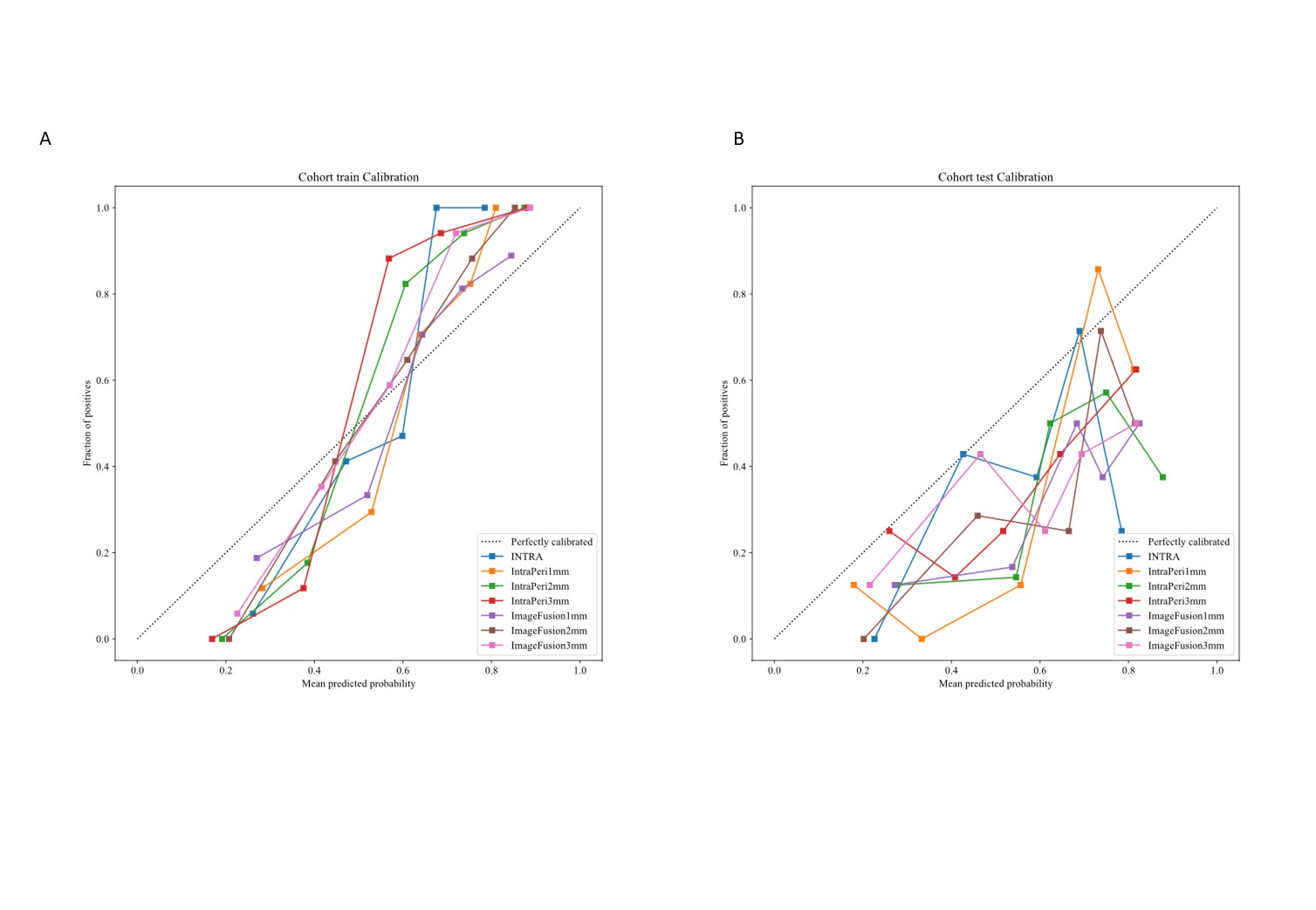

Supplement: Supplementary file 4 — Supplementary Material 4 [file 12880_2025_1790_MOESM4_ESM.jpg]
